# MR elastography as a method to estimate aortic stiffness and its comparison against MR based pulse wave velocity measurement

**DOI:** 10.1186/1532-429X-15-S1-P240

**Published:** 2013-01-30

**Authors:** Anirudh Damughatla, Brian Raterman, Orlando P Simonetti, Travis Sharkey-Toppen, Ning Jin, Richard D White, Arunark Kolipaka

**Affiliations:** 1 Biomedical Engineering, The Ohio State University, Columbus, OH, USA; 2 Radiology, The Ohio State University Wexner Medical Center, Columbus, OH, USA; 3 Internal Medicine, The Ohio State University Wexner Medical Center, Columbus, OH, USA; 4 Siemens Medical Solutions Inc., Columbus, OH, USA

## Background

Arterial (aortic) stiffness is a well-recognized pathophysiological change that plays a significant role in the determination of risk factors for various cardiovascular diseases[1]. Measurement of arterial stiffness using pulse wave velocity (PWV) is the gold standard among non-invasive modalities. Recently, a novel non-invasive MRI based technique known as magnetic resonance elastography (MRE) was developed to determine the stiffness of the aorta[2]. The aim of the study is to compare the abdominal aortic stiffness obtained using MRI based PWV stiffness measurements against MRE based stiffness measurements.

## Methods

In-vivo aortic MRE and MRI was performed on 8 healthy volunteers (Ages 18-35yrs). All imaging was performed using a 3T-MRI Scanner (Tim-Trio, Siemens Healthcare, Germany). The volunteers were positioned in the supine position and placed head first in the scanner. 60Hz mechanical waves were introduced in to the aorta using a pneumatic diver[2]. A GRE-MRE and phase contrast (PC)-MRI sequences were performed to obtain wave and velocity data on a sagittal slice of the aorta. The imaging parameters for MRE included: TE/TR=21.3/25ms, matrix =128x64, FOV=40cm, α =16, and a motion encoding gradient of 60Hz was applied separately in the x, y, and z direction to encode motion. The imaging parameters for the PC-MRI included: TE/TR=2.1/9.15ms, venc=150,175cm/s; matrix=192x144, FOV=30x40cm2, α =15, #cardiac phases=128. The sagittal images were masked to obtain the major portion of the aorta for both MRE and PC-MRI data analysis. Then, MRE wave images were analyzed using MRE-Lab (Mayo Clinic Rochester, MN) to obtain the stiffness of the aorta[3]. PC-MRI phase images were analyzed using in house custom built software in Matlab (Mathworks, Natic, MA) to obtain the PWV measurements, and the stiffness was calculated by using Moens-Korteweg equation[4].

## Results

**Figure 1:**
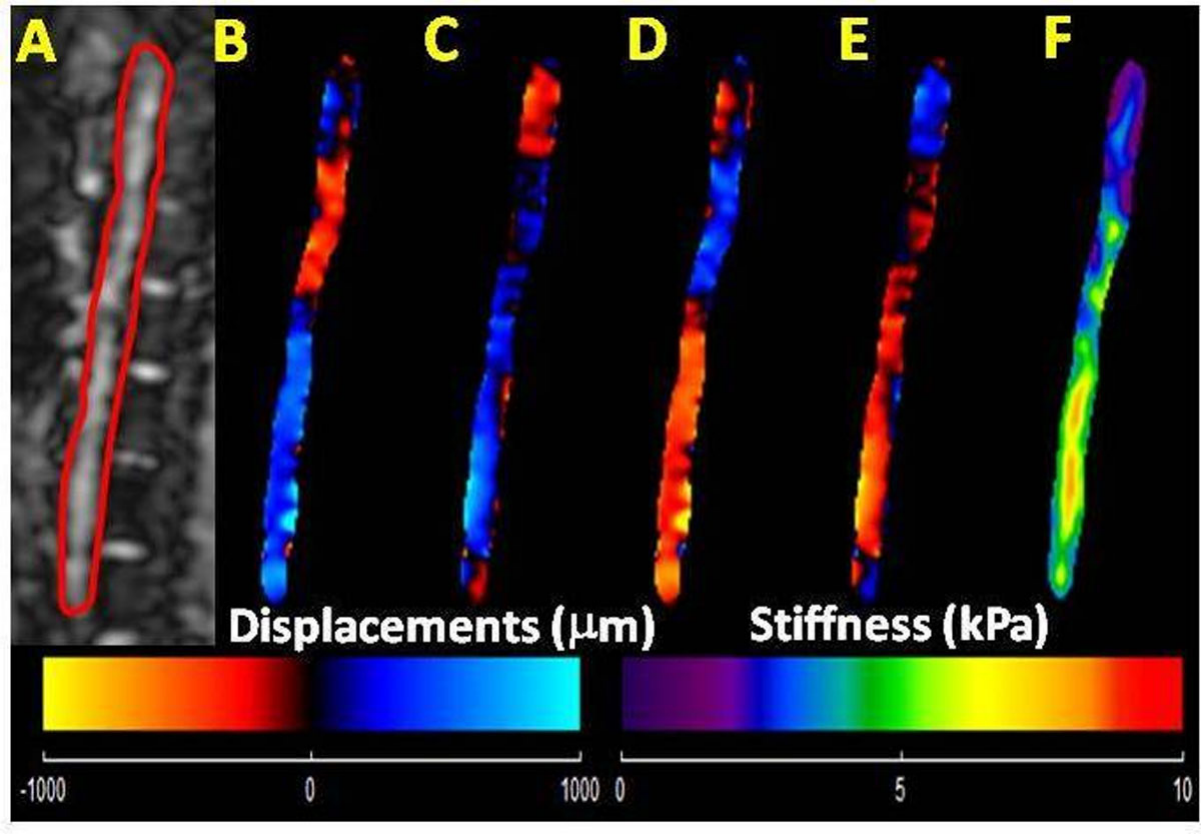
Healthy Volunteer. A: Sagittal magnitude image with contour (red line) delineating abdominal aorta. B-E: Snap shot of four phases of propagating waves. F: The stiffness map from x, y, and z encoding directions with a mean stiffness of 5.5 kPa.

**Figure 2:**
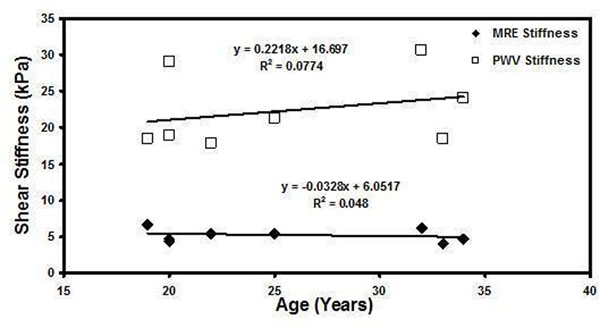
Plot of shear stiffness obtained using MRE and PWV as a function of age.

Figure 1A-E shows the sagittal magnitude image with the contours used for segmenting the abdominal aorta and corresponding snap shots of wave propagation in one of the volunteers. Figure 1F shows the weighted stiffness map from 3 encoding directions with a mean stiffness value of 5.5±1.3kPa. Figure 2 shows the plot of stiffness values obtained using MRE and PWV Vs. age indicating no trend in this data set. Furthermore, the MRE stiffness (range 4-6.7kPa) and PWV (range 4.2-5.2m/s) values obtained from all volunteers were in the normal range[2,5].

## Conclusions

This study demonstrated the feasibility of comparing MRE based stiffness estimates and PWV based stiffness estimates in the same imaging plane of the aorta.

## Funding
